# Therapeutic Progress

**Published:** 1889-12

**Authors:** H. H. Haralson

**Affiliations:** Forest, Miss.


### THERAPEUTIC PROGRESS.

H. H. HARALSON, M. D., FOREST, MISS.

In reviewing the history of therapeutics, even from its remotest existence down to the present time, we are unable to find a period in which so many new remedies were presented to the profession as now. While this is a fact it is equally true that actual progress in this department of our science is very slow.

It is said that the average length of human life is increasing. The truthfulness of this statement we are not prepared to question, but though we allow the correctness of it it does not necessitate an admittance of therapeutic progress. Measures to prevent disease or prophylaxis both in place and person ranks first as an instrument of lengthening human life. We have more confidence in our means to this end than we have in the application of medicine to disease or therapeutics proper. Prophylaxis is saving hundreds and thousands of human lives annually. We have but to look only a few months back and we cannot fail to see what a judicious system of prophylaxis did for this city and the State of Mississippi. Again, that "let alone" system, that intelligent forbearance in the treatment of disease, has accomplished much, and if we had any way of estimating the lives saved by it we doubt not that it would astonish the world.

In investigating this subject we might take up a few of the leading diseases and see if any improvement has been made in their treatment during the last few years. Do we know anything more about the treatment of syphilis than a few years back? Can we treat malarial troubles more satisfactorily than a decade ago? Quinine, I believe, is claimed to be a specific in malaria. Patients sometimes die with it, nevertheless, though they may have taken immense quantities of it.

We have made, during the last quarter of a century, some improvements in the treatment of typhoid fever and pneumonia, but not by the application of medicine to the disease. We have learned to let them alone, or, in other words, to practice a judicious forbearance. I do not believe that we possess a single medicine that will shorten, in the least degree, the course of typhoid fever.

Now let us take up some of the new remedies of the present day or of this decade. To speak of all of them would require too much time, or even of all the leading ones, so we will investigate briefly only a few of them. When two men, equally learned and wise in science, differ materially concerning the action of a certain drug, and when that difference of opinion is the result of honest and careful investigation, that drug has no exact place in the science of medicine or as a therapeutic agent. Where an overwhelming majority of the profession get similar results on the human organism from a certain medicine then we can ascribe to that drug an exact place in science. Every physician in this hall might administer Chloroform to as many different persons, and the result, insensibility, would be obtained on each. We know then exactly where to place it. We know that it is a general anæsthetic. The same may be said of Quinine as an antiperiodic, of Opium as an anodyne, and of Ergot as an oxytocic. Now how is it with our so-called new remedies?

Take Cocaine. To what place do you propose to ascribe it? Is it a local anæsthetic? A few years ago the profession was wild, yes, absolutely so, on Cocaine as a local anæsthetic. Enconiums of praise from every part of the civilized world were lavished upon it as such. It was sold at fabulous prices. If a person dared say anything against it he was pronounced a monstrous ignoramus. To-day its properties as an important local anæsthetic are honestly and truthfully questioned by a respectable number of men of no mean scientific attainments. It is strange indeed that there should be any doubt at all about a medicine of this kind. How many capital operations would be required to establish, beyond the peradventure of doubt, the reputation of Chloroform as a general anæsthetic. One, only one, would be sufficient to convince the most skeptical. If Cocaine, then, absolutely possesses such properties, why is it that it takes so long to place it on a sure footing in the confidence of observing scientific men ?

Let us examine the testimony of observers on the physiological and therapeutical action of Antifebrine. One observer claims that it never produces cynanopathy, another that it does, and in consequence of which regards it as a dangerous drug. One claims that by its continuous use the patient grows accustomed to it and its danger lessened, another that patients do not become inured to its use though it may be continued for quite a time. One observer avers that the fall of temperature is noticeable in one hour after its administration, others not earlier than three and from that up to eight. Some have observed it produce profuse diuresis and diaphoresis, another has never witnessed such results. One observer gave eight grains six different times, with no reduction of temperature in a case of pneumonia, while two four grain doses of Thalline reduced the temperature four degrees. Our own observation, which is not very extensive, is that it possesses no advantages as an antipyretic over those of long use and standing.

We have used Antipyrine more frequently than Antifebrine, and it has been satisfactorily demonstrated to us that it is a very inferior remedy. In rheumatism we are fully satisfied that no good results from its use. We have used it many times in this disease and in every instance have utterly failed to benefit our patient. I do not believe that it can be pushed with safety to the patient to such an extent that a reduction of temperature can be had in rheumatism of an inflammatory character. I do not know that I have ever injured a patient by its use. I am quite sure, however, that no accident of a serious character has ever followed its administration in my hands. As to the reports on this drug we find them as conflicting as those on Antifebrine—

all admitting, however, that alarming symptoms often follow its administration. Nearly all observers have had gastric irritation follow its use. We have never had any trouble from this source. Some claim that it possesses anodyne properties, others that it is a signal failure as such. We have often seen happy effects from its administration in headache and we have as often seen it fail to produce any beneficial effect. Upon the whole, we do not believe it is a very great acquisition to the list of therapeutic agencies.

We might review the whole list of new remedies and we would find reports on them no more favorable than those above mentioned.

Though we have for centuries groped our way in the dark with no safer guide than empiricism, we are driven to conclude and assert that there must be some cause for these conflicting reports. Much of it we are forced to admit is due to a lack of prudence on the part of those who practice experimental observation.

We are persuaded that one of the most serious errors made by observers in the cure of disease with new remedies is in their diagnosis. Let me have the naming of a disease without criticism and I can take almost any medicine and perform wonderful feats of cures with it. How often do we see long reports of cures of membranous croup with sulph. of Copper or sub sulph. of Mercury, or something else that would have as little beneficial effect. Our opinion is, that there is no medicine known to our profession that in the very least degree checks the ravages of this fearfully fatal disease. If so we are free to confess that we have never been so fortunate as to find it, though we have hunted it long and faithfully. You also see reports of cures of Bright's disease, diagnosticated perhaps by pains in the back. A disease so fatal as this cured! Case after case cured, and why? Because the observer had the naming of the disease without criticism.

You might base a plan of treatment on a number of cases of typhoid fever terminating favorably in from eighteen to twenty-four days and it would prove nothing positively. It might be of some value negatively, because it could have been possible for them to have been worse, but as positive evidence it would be worth absolutely nothing.

The earliest observations of a new medicine are always favorable, though later ones may be very unfavorable. This is necessarily the result of errors in diagnosis and hasty conclusions. In our eagerness to present something new we overlook its failures, though numerically they may be of more value in arriving at truthful conclusions than its successes.

During the long centuries of our existence as a profession we have fallen into many errors; we have found few truths. We have witnessed seeming gem after gem of truth vanish from our view. We have seen that which past generations regarded as truth tramped in the dust by the next. We are not unmindful of the contradictions of the past, of the incongruities of the present. Under such circumstances we look around us for a place of safety and are almost driven to seek it in nihilism. So many ages and ages have been spent in its study; so many brilliant minds have been consumed, offered as sacrifices upon its altar, and yet so little progress has been made. With an experience of two thousand years, and what do we know? Echo answers, what do we know?

[We have only to add that the thanks of the Mississippi State Medical Society were voted Dr. Haralson for the above paper!!—Editors.]

